# Diagnostic yield and clinical relevance of expanded genetic testing for cancer patients

**DOI:** 10.1186/s13073-022-01101-2

**Published:** 2022-08-15

**Authors:** Ozge Ceyhan-Birsoy, Gowtham Jayakumaran, Yelena Kemel, Maksym Misyura, Umut Aypar, Sowmya Jairam, Ciyu Yang, Yirong Li, Nikita Mehta, Anna Maio, Angela Arnold, Erin Salo-Mullen, Margaret Sheehan, Aijazuddin Syed, Michael Walsh, Maria Carlo, Mark Robson, Kenneth Offit, Marc Ladanyi, Jorge S. Reis-Filho, Zsofia K. Stadler, Liying Zhang, Alicia Latham, Ahmet Zehir, Diana Mandelker

**Affiliations:** 1 Department of Pathology and Laboratory Medicine, Memorial Sloan Kettering Cancer Center, New York, NY USA; 2 Sloan Kettering Institute, Memorial Sloan Kettering Cancer Center, New York, NY USA; 3 Department of Medicine, Memorial Sloan Kettering Cancer Center, New York, NY USA; 4 Present Address: Department of Pathology and Laboratory Medicine, David Geffen School of Medicine, University of California, Los Angeles (UCLA), Los Angeles, CA USA; 5 Present Address: Precision Medicine and Biosamples, Oncology R&D, AstraZeneca, New York, NY USA

## Abstract

**Background:**

Genetic testing (GT) for hereditary cancer predisposition is traditionally performed on selected genes based on established guidelines for each cancer type. Recently, expanded GT (eGT) using large hereditary cancer gene panels uncovered hereditary predisposition in a greater proportion of patients than previously anticipated. We sought to define the diagnostic yield of eGT and its clinical relevance in a broad cancer patient population over a 5-year period.

**Methods:**

A total of 17,523 cancer patients with a broad range of solid tumors, who received eGT at Memorial Sloan Kettering Cancer Center between July 2015 to April 2020, were included in the study. The patients were unselected for current GT criteria such as cancer type, age of onset, and/or family history of disease. The diagnostic yield of eGT was determined for each cancer type. For 9187 patients with five common cancer types frequently interrogated for hereditary predisposition (breast, colorectal, ovarian, pancreatic, and prostate cancer), the rate of pathogenic/likely pathogenic (P/LP) variants in genes that have been associated with each cancer type was analyzed. The clinical implications of additional findings in genes not known to be associated with a patients’ cancer type were investigated.

**Results:**

16.7% of patients in a broad cancer cohort had P/LP variants in hereditary cancer predisposition genes identified by eGT. The diagnostic yield of eGT in patients with breast, colorectal, ovarian, pancreatic, and prostate cancer was 17.5%, 15.3%, 24.2%, 19.4%, and 15.9%, respectively. Additionally, 8% of the patients with five common cancers had P/LP variants in genes not known to be associated with the patient’s current cancer type, with 0.8% of them having such a variant that confers a high risk for another cancer type. Analysis of clinical and family histories revealed that 74% of patients with variants in genes not associated with their current cancer type but which conferred a high risk for another cancer did not meet the current GT criteria for the genes harboring these variants. One or more variants of uncertain significance were identified in 57% of the patients.

**Conclusions:**

Compared to targeted testing approaches, eGT can increase the yield of detection of hereditary cancer predisposition in patients with a range of tumors, allowing opportunities for enhanced surveillance and intervention. The benefits of performing eGT should be weighed against the added number of VUSs identified with this approach.

**Supplementary Information:**

The online version contains supplementary material available at 10.1186/s13073-022-01101-2.

## Background

Germline genetic testing (GT) for hereditary cancer predisposition has become increasingly important in the management of cancer patients [1, 2]. Identifying patients with hereditary predisposition can inform targeted therapies for certain cancers and allow for timely surveillance and preventative interventions for both patients and at-risk family members [3–7]. Traditionally, testing for cancer predisposition heavily relied on clinical criteria from national guidelines to select the most clinically appropriate genes based on the patient’s prior probability of carrying a germline alteration dictated by their tumor type, age of onset, and/or family histories [8–10]. More recently, broader gene panels are used by many clinicians for patients with a wide range of cancer histories. Expanded GT (eGT) without preselection of patients or genes uncovered hereditary cancer predisposition in a greater proportion of patients than previously anticipated, including those who do not meet the current testing criteria [11–21]. We previously demonstrated that 17% of 1040 advanced cancer patients receiving eGT harbored pathogenic or likely pathogenic (P/LP) germline variants in cancer predisposition genes. Additionally, 56% of these findings would have not been identified via guideline-based targeted GT at the time, as the patients did not meet the criteria to receive traditional GT for these genes. Additional studies have also demonstrated that guideline-based GT failed to detect a significant portion of patients with germline alterations [11–21]. Reasons for restricting GT to selected genes include the uncertain clinical utility of identifying P/LP variants in genes outside the recommended ones based on established guidelines and the potential burden of variants of uncertain significance (VUSs). To explore the diagnostic yield and utility of eGT in patients with a broad range of solid tumors, we analyzed the eGT results in a cohort of 17,523 cancer patients who received paired tumor-normal sequencing over a 5-year period at a tertiary cancer hospital. Additionally, for 9187 of the patients with five common cancers frequently interrogated for hereditary predisposition (breast, colorectal, ovarian, pancreatic, and prostate cancer), we assessed the clinical implications of genes not typically targeted for their cancer type.

## Methods

### Patient cohort

The patient cohort consisted of 17,523 patients diagnosed with a broad range of solid tumors unselected for current GT criteria such as cancer type, age of onset, and/or family history of disease, who were treated at Memorial Sloan Kettering (MSK) Cancer Center (MSKCC) and prospectively consented to germline analysis as part of the MSK Integrated Mutation Profiling of Actionable Cancer Targets (MSK-IMPACT; ClinicalTrials.gov identifier, NCT01775072) paired tumor-blood DNA sequencing test between July 2015 and April 2020. Patients with cancer consenting to tumor sequencing for somatic profiling were offered participation in the MSK-IMPACT germline study by their treating physicians at MSKCC. Pre-test genetic counseling was provided using a video consent explaining the risks and benefits of testing for inherited variants. Eligibility was open to all cancer patients regardless of cancer type diagnosis or family history but was restricted to those who also consented to matched tumor sequencing. Peripheral blood samples were collected from the participants for GT. The study cohort included patients with the following cancer types: breast cancer (*n* = 2243), prostate cancer (*n* = 2114), colorectal cancer (*n* = 2060), pancreatic cancer (*n* = 1648), endometrial cancer (*n* = 1191), ovarian cancer (*n* = 1122), bladder cancer (*n* = 838), esophagogastric carcinoma (*n* = 661), renal cell carcinoma (*n* = 592), glioma (*n* = 499), soft tissue sarcoma (*n* = 433), biliary cancer (*n* = 410), melanoma (*n* = 332), non-small cell lung cancer (*n* = 213), embryonal tumor (*n* = 186), thyroid cancer (*n* = 153), mesothelioma (*n* = 145), appendiceal cancer (*n* = 133), cervical cancer (*n* = 122), germ cell tumor (*n* = 119), hepatocellular carcinoma (*n* = 106), uterine sarcoma (*n* = 102), osteosarcoma (*n* = 96), gastrointestinal stromal tumor (*n* = 85), gastrointestinal neuroendocrine tumor (*n* = 81), non-melanoma skin cancer (*n* = 77), small bowel cancer (*n* = 73), head and neck carcinoma (*n* = 71), cancer of unknown primary (*n* = 506), others (*n* = 1112). All patients were tested for 76 or 88 hereditary cancer predisposition genes on MSK-IMPACT under an institutional review board-approved protocol (please see Additional file 1: Table S1 for the list of genes) [15, 22, 23]. Genetic testing reports were issued to the medical record, and individuals with P/LP variants were invited for genetic counseling. The results from eGT of 9187 patients with five cancer types frequently interrogated in traditional guideline-based GT (breast, colorectal, ovarian, pancreatic, and prostate cancer) were further analyzed to assess the yield in genes that have been associated with their cancer type and the clinical implications of other genes not typically targeted for their disease. All patients provided written, informed consent for GT.

### Genetic testing and analysis

The MSK-IMPACT germline analysis is a New York State Department of Health-approved assay and was performed in our CLIA-approved laboratory using next-generation sequencing on DNA isolated from the blood, as described previously [15]. Briefly, DNA was isolated from peripheral blood specimens using Chemagic STAR DNA Blood-400 kits (PerkinElmer). MSK-IMPACT, a hybridization capture-based next-generation sequencing assay based on custom-designed biotinylated probes (NimbleGen) [22, 24], was used for library preparation. Captured DNA fragments were sequenced on an Illumina HiSeq 2500 as paired-end 100-bp reads. Variants were called using MuTect [25] and Genome Analysis Toolkit (GATK) Haplotypecaller [26] and were filtered based on 25% variant allele fraction for single nucleotide variants (SNVs) and 15% for insertions/deletions (indels) and 20× coverage thresholds. All variants with < 1% population frequency in the Genome Aggregation Database (gnomAD) [27] were reviewed and interpreted. Copy number variants (deletions and duplications of single or multiple exons) in the target genes were captured and assessed using a validated in-house developed pipeline [15, 24]. Variants, including single nucleotide variants, small deletions and/or insertions, and copy number variants, were interpreted and classified by clinical molecular geneticists and molecular genetic pathologists based on the American College of Medical Genetics and Genomics (ACMGG) criteria [28]. Identification of a pathogenic or likely pathogenic (P/LP) variant was considered as a positive result. Variants internally classified as VUS were not reported. Clinical impact of P/LP variants was assessed based on management guidelines from the National Comprehensive Cancer Network (NCCN) [8, 9, 29, 30] (summarized in Additional file 2: Table S2).

Genes were grouped based on their penetrance and inheritance type (Additional file 3: Table S3). Five specific variants or variant types were considered as having a different penetrance or inheritance pattern compared to the typical pathogenic variants in the respective genes: *APC* p.Ile1307Lys having low penetrance [31], *CHEK2* p.Ile157Thr having uncertain penetrance [32, 33], *EGFR* loss-of-function variants having autosomal recessive (AR) inheritance for neonatal ectodermal dysplasia with severe skin defects and gastrointestinal dysfunction and uncertain risk for lung cancer [34, 35], *FH* p.Lys477dup having AR inheritance for fumarate hydratase deficiency with uncertain risk for hereditary leiomyomatosis and renal cell cancer (HLRCC) [36], and *VHL* p.Arg200Trp having AR inheritance for Chuvash polycythemia and uncertain risk for von Hippel-Lindau syndrome [37, 38]. Confidence intervals (95%CI) were calculated based on sample sizes using the Wilson/Brown method.

## Results

### Rate of hereditary cancer predisposition identified in eGT of patients with solid tumors

**Fig. 1:**
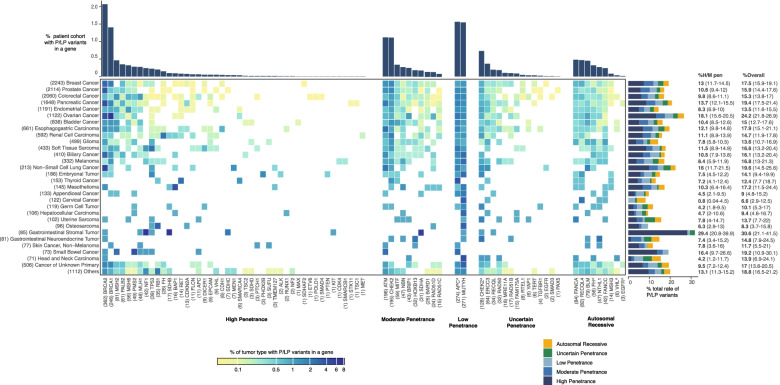
Distribution of pathogenic/likely pathogenic variants identified in eGT of 17,523 cancer patients across genes and cancer types. The distribution of P/LP variants identified in patients with each cancer type, grouped based on gene penetrance and inheritance pattern, is presented. The number of patients in each category is in parentheses. Specific variants that show different penetrance or inheritance pattern from typical variants in the gene were plotted separately: APC^: *APC* p.Ile1307Lys; CHEK2^: *CHEK2* p.Ile157Thr; FH^: *FH* p.Lys477dup; and VHL^: *VHL* p.Arg200Trp. Percentage of high/moderate-penetrance (%H/M pen) variants identified in each cancer type and overall percentage of patients with P/LP variants (%Overall) are presented with 95% confidence intervals in parentheses

The patient cohort consisted of 17,523 patients with a broad range of solid tumors who received eGT. In comparison with the incidence rates reported by the National Cancer Institute Surveillance, Epidemiology, and End Results Program [39], our cohort was particularly enriched for pancreatic, ovarian, endometrial/cervical, CNS cancers, and sarcomas, while having a relatively lower proportion of lung, head/neck, thyroid, breast cancers, and melanomas (Additional file 4: Fig. S1). P/LP variants were identified in 16.7% (2930/17,523) (95%CI 16.2–17.3%) of patients overall, with 10.6% (1865/17,523) (95%CI 10.2–11.1%) having P/LP variants in a high- or moderate-penetrance gene with autosomal dominant inheritance (Fig. 1). In cancer types with > 1000 patients tested, ovarian cancer had the highest rate of patients with P/LP variants (24.2%), followed by pancreatic cancer (19.4%) and breast cancer (17.5%), with 18.1%, 13.7%, and 13% having P/LP variants in high/moderate-penetrance genes, respectively. In other cancer types represented by a smaller number of patients in our eGT cohort, the highest rates of germline P/LP variants were identified in gastrointestinal stromal tumors (30.6%), non-small cell lung cancer (19.6%), small bowel cancer (19.2%), esophagogastric cancer (17.9%), and mesotheliomas (17.2%), with 29.4%, 16%, 12.1%, 16.4%, and 11.6% having P/LP variants in high/moderate-penetrance genes, respectively (Fig. 1).

Positive results include findings in genes that are known to be associated with the patient’s cancer type and those in genes that have not been associated with the patient’s current disease, which likely represent secondary findings. P/LP variants in genes that confer increased risk for the individual’s tumor type were also identified in patients with cancer types that are not frequently interrogated in traditional targeted GT models, such as 8.2% (6/73) of small bowel cancer patients having *MLH1*, *MSH2*, or *PMS2* [40], 4.1% (6/145) of mesothelioma patients having *BAP1* [41], 3.1% (3/96) of osteosarcoma patients having *RB1* [42], and 2.5% (11/433) of soft tissue sarcoma patients having *TP53* [43–45] P/LP variants.

A significant proportion of our cohort (1.2%) had one of the three *BRCA1/BRCA2* Ashkenazi Jewish founder variants [46–48], due to the prevalence of individuals with Ashkenazi Jewish ancestry in our patient population (16% of patients receiving MSK-IMPACT [49]).

### Diagnostic yield of eGT for breast, colorectal, ovarian, pancreatic, and prostate cancer

**Fig. 2:**
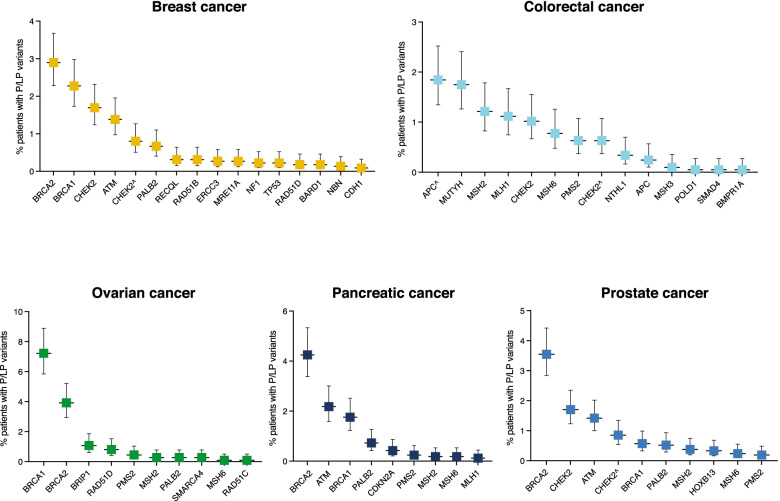
Rate of positive results identified in each gene in patients with five common cancer types. Percentage of patients with breast cancer (*n* = 2243), colorectal cancer (*n* = 2060), ovarian cancer (*n* = 1122), pancreatic cancer (*n* = 1648), and prostate cancer (*n* = 2114) who had P/LP variants in genes that have been associated with each cancer type. Error bars represent 95% confidence intervals

For patients with breast, colorectal, ovarian, pancreatic, and prostate cancer, GT is often pursued in a guideline-dependent manner, either by targeting a group of genes that are strongly associated with the particular cancer type or testing larger panels of hereditary cancer predisposition genes including those that are not known to increase the risk for the patient’s current disease. We assessed the rate of positive results (identification of P/LP variants) in each gene for these five common cancers that are most frequently interrogated for hereditary predisposition in the current practice and evaluated the rate of additional findings in genes that are not known to be associated with the patient’s cancer type (Fig. 2).

In breast cancer patients (*n* = 2243), the overall yield of eGT was 17.5% (392/2243). *BRCA1* and *BRCA2* P/LP variants were identified in 2.9% (*n* = 51) and 2.3% (*n* = 65) of the patients, respectively, and accounted for 26.9% of all positive results in these patients. Three other commonly targeted genes, *CHEK2*, *ATM*, and *PALB2* [29, 50–52], had a diagnostic yield of 2.5% (*n* = 56), 1.4% (*n* = 31), and 0.7% (*n* = 15), respectively. High-penetrance genes that implicate breast cancer risk and are often targeted in the presence of additional features in the patient’s personal and/or family history [29, 50–52] and had positive results in our cohort include *NF1* with 0.2% (*n* = 5), *TP53* with 0.2% (*n* = 5), and *CDH1* with 0.09% (*n* = 2) yield. While these three genes added a minor increase in the diagnostic yield, all five patients with *NF1* variants had features of neurofibromatosis type 1, both of the two patients with *CDH1* variants had lobular breast carcinoma, and one of the two patients with *TP53* variants had a history of sarcoma and breast cancer at 29 years of age. One patient with the *TP53* variant had breast cancer at 44 years of age and did not meet the current *TP53* GT criteria [43, 53]. Other genes with moderate, low, or uncertain penetrance that have been implicated in breast cancer [29, 50–52, 54–56], *RECQL*, *RAD51B*, *ERCC3*, *MRE11A*, *RAD51D*, *BARD1*, and *NBN*, had a yield of 0.3% (*n* = 7), 0.3% (*n* = 7), 0.3% (*n* = 6), 0.3% (*n* = 6), 0.2% (*n* = 4), 0.2% (*n* = 4), and 0.1% (*n* = 3), respectively.

In colorectal cancer patients (*n* = 2060), the overall yield of eGT was 15.3% (316/2060). The highest rate of positive results was in *APC*, with the low-penetrance p.Ile1307Lys variant identified in 1.8% (*n* = 38) and other *APC* variants in 0.2% (*n* = 5), followed by monoallelic *MUTYH* variants in 1.7% (*n* = 36), and Lynch syndrome-associated variants in *MSH2*, *MLH1*, *MSH6*, and *PMS2* [30] identified in 1.2% (*n* = 25), 1.1% (*n* = 23), 0.8% (*n* = 16), and 0.6% (*n* = 13) of the patients, respectively. P/LP variants in other genes that have been associated with colorectal cancer [30, 57], *CHEK2*, *NTHL1* (monoallelic variants), *MSH3* (monoallelic variants), *POLD1*, *BMPR1A*, and *SMAD4*, were identified in 1.6% (*n* = 34), 0.3% (*n* = 7), 0.1% (*n* = 2), 0.05% (*n* = 1), 0.05% (*n* = 1), and 0.05% (*n* = 1) of the patients, respectively. Of note, the *POLD1* carrier had hyper-mutated colon adenocarcinoma, the *BMPR1A* carrier had a hamartomatous polyp, and the *SMAD4* carrier had a history of a juvenile polyp, consistent with the identified genes, although the patients with *BMPR1A* and *SMAD4* variants do not meet the current GT criteria for the respective genes [30].

In ovarian cancer patients (*n* = 1122), the overall yield of eGT was 24.2% (272/1122). *BRCA1* and *BRCA2* P/LP variants were identified in 7.2% (*n* = 81) and 3.9% (*n* = 44) of the patients and accounted for 42% of all positive results in these patients. Other genes implicated in ovarian cancer [29, 58–60], *BRIP1*, *RAD51D*, *PALB2*, and *RAD51C*, had a yield of 1.1% (*n* = 12), 0.8% (*n* = 9), 0.3% (*n* = 3), and 0.09% (*n* = 1), respectively. *MSH2*, *PMS2*, and *MSH6* variants were identified in 0.3% (*n* = 3), 0.5% (*n* = 5), and 0.09% (*n* = 1), with a total of 0.9% of ovarian cancer patients having Lynch syndrome-associated variants, and 78% (7/9) of them had endometrioid, clear cell, or mixed ovarian carcinoma/carcinosarcoma, whereas two had high-grade serous ovarian carcinoma [61, 62]. Microsatellite instability (MSI) and/or loss of the mutated protein’s expression by immunohistochemistry (IHC) in the tumors were detected in five patients, who were considered to meet Lynch syndrome GT criteria based on their MSI/mismatch repair-deficient tumor profiles [62], whereas four patients with *MSH2* or *PMS2* variants had microsatellite stable/indeterminate tumors with retained mismatch repair protein expression. Additionally, *SMARCA4* variants were identified in three patients with small cell carcinoma of the ovary, hypercalcemic type, accounting for 0.3% of our ovarian cancer patient cohort.

In pancreatic cancer patients (*n* = 1648), the overall yield of eGT was 19.4% (319/1648). *BRCA2*, *ATM*, and *BRCA1* [29] variants were identified in 4.2% (*n* = 70), 2.2% (*n* = 36), and 1.8% (*n* = 29) of the patients, respectively. *PALB2* and *CDKN2A* [29] had a yield of 0.7% (*n* = 12) and 0.4% (*n* = 7), respectively. Variants in *PMS2*, *MSH2*, *MSH6*, and *MLH1* [29] were identified in 0.2% (*n* = 4), 0.2% (*n* = 3), 0.2% (*n* = 3), and 0.1% (*n* = 2), respectively, with 0.7% of pancreatic cancer patients having Lynch syndrome-associated variants overall.

In prostate cancer patients (*n* = 2114), the overall yield of eGT was 15.9% (337/2114). *BRCA2*, *CHEK2*, *ATM*, *BRCA1*, *PALB2*, and *HOXB13* [63] variants were identified in 3.5% (*n* = 75), 2.5% (*n* = 54), 1.4% (*n* = 30), 0.6% (*n* = 12), 0.5% (*n* = 11), and 0.3% (*n* = 7) of the patients, respectively. Additionally, *MSH2*, *MSH6*, and *PMS2* [63] variants were identified in 0.4% (*n* = 8), 0.2% (*n* = 5), and 0.2% (*n* = 4), with 0.8% of prostate cancer patients having Lynch syndrome-associated variants overall.

#### Additional findings discovered in eGT

**Fig. 3:**
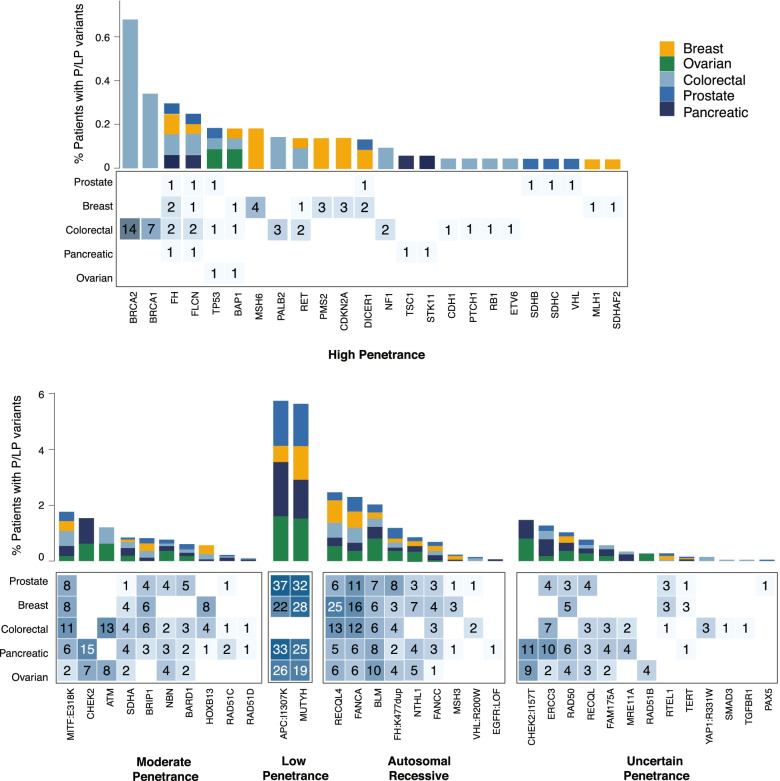
Pathogenic/likely pathogenic variants identified in genes not associated with the patient’s cancer type. Genes were grouped based on inheritance pattern, and autosomal dominant genes were further grouped based on penetrance as high, moderate, low, and uncertain. In three genes, only specific variants were targeted: *HOXB13* p.Gly84Glu, *MITF* p.Glu318Lys, and *YAP1* p.Arg331Trp. Certain variants were considered as having different penetrance or inheritance pattern from typical variants in the gene: *APC* p.Ile1307Lys and *CHEK2* p.Ile157Thr as having uncertain penetrance; *FH* p.Lys477dup, *VHL* p.Arg200Trp, and *EGFR* loss-of-function variants as being AR. The percentage of carriers within each cancer type is presented in the upper panels. The number of P/LP variants identified in each gene and cancer type are presented in the lower panels

For individuals with breast, colorectal, ovarian, pancreatic, and prostate cancer, we next sought to characterize the additional P/LP variants in genes other than those that are associated with the patient’s current cancer type, as described above. Overall, 765 additional P/LP variants in genes not known to be associated with the patient’s current cancer type were identified in 8% (736/9187) of the patients with five common cancer types, with 0.3% (29/9187) having multiple such variants (Fig. 3). Additional findings were identified in 7% (156/2243) of breast, 6.8% (140/2060) of colorectal, 11.2% (125/1122) of ovarian, 10% (164/1648) of pancreatic, and 7.2% (151/2114) of prostate cancer patients. Additionally, 1.7% of breast, 1.5% of colorectal, 2.2% of ovarian, 1.4% of pancreatic, and 1.1% of prostate cancer patients had multiple P/LP variants identified in eGT, including those in genes that are associated with their cancer type.

Overall, 3.3% (299/9187) of patients had an additional finding that indicated early or additional surveillance, and 0.2% (17/9187) had a finding that indicated prophylactic surgery recommendations to reduce future cancer risks for the patient and their carrier family members (Fig. 3, Additional file 2: Table S2). Monoallelic variants in AR genes conferring carrier status, which are not expected to increase disease risk but may have reproductive planning implications, were identified in 3% (278/9187) of the patients.

A total of 69 patients (0.8%) had a P/LP variant in a high-penetrance gene that is not associated with their cancer type (Table 1). We retrospectively reviewed the detailed clinical and family histories of these patients to assess whether they had any clinical features or history that was consistent with these findings and if they met the traditional GT criteria for the identified genes per current NCCN guidelines. Of the 69 patients, 18 (26%) met the current criteria to receive GT for the additional gene identified in eGT based on their personal and/or family histories. These include four colorectal cancer patients with *BRCA1/BRCA2* and a history of breast cancer, one breast cancer patient with *MLH1* and a history of endometrial cancer, one colorectal cancer patient with *RB1* and a history of retinoblastoma, one colorectal cancer patient with *NF1* and features of neurofibromatosis type 1, one prostate cancer patient with *FLCN* and fibrofolliculomas and lung cysts, and one pancreatic cancer patient with *TSC1* and angiomyolipoma, brain lesions, and bilateral renal cysts, which were discovered upon receiving eGT results (Table 1). Nine patients met the GT criteria based on their family histories.

**Table 1:** Additional high-penetrance P/LP variants identified via eGT in genes not associated with the patient’s cancer type

Pt #	Gender	Cancer Dx at the time of testing	Age range at Dx	Additional high-penetrance gene identified	Meets the GT criteria for the additional gene?	Other genes identified in eGT	Other Hx of cancer	OncoKB classification	Future cancer risk management implications
1	F	Colorectal	60s	BRCA2	Y		Breast	3B	Surveillance and prophylactic surgery
2	F	Colorectal	70s	BRCA2	Y		Breast, lung, sarcoma, skin	3B	Surveillance and prophylactic surgery
3	M	Prostate	50s	FLCN	Y	CHEK2		NA	Surveillance
4	F	Colorectal	80s	ETV6	N		Breast, kidney, chronic lymphocytic leukemia	3B	Surveillance
5	F	Breast	60s	MSH6	N		Breast	NA	Surveillance
6	F	Colorectal	60s	BRCA1	Y		Breast	3B	Surveillance and prophylactic surgery
7	M	Colorectal	50s	BRCA2	Y	FANCC		3B	Surveillance
8	F	Breast	60s	FH	N			NA	Surveillance
9	F	Breast	40s	CDKN2A	N		Melanoma	NA	Surveillance
10	F	Breast	30s	CDKN2A	N			NA	Surveillance
11	F	Breast	50s	MSH6	N			NA	Surveillance
12	M	Prostate	50s	VHL	N			NA	Surveillance
13	F	Breast	40s	FH	N	CHEK2		NA	Surveillance
14	M	Prostate	60s	SDHB	N			NA	Surveillance
15	F	Colorectal	50s	BRCA2	Y		Vulva	3B	Surveillance and prophylactic surgery
16	F	Breast	30s	MLH1	Y		Uterus	1*	Surveillance
17	F	Breast	40s	PMS2	N			NA	Surveillance
18	F	Colorectal	50s	CDH1	N			NA	Surveillance and prophylactic surgery
19	F	Pancreas	60s	FLCN	N			NA	Surveillance
20	M	Colorectal	70s	FLCN	N	CHEK2	Prostate	NA	Surveillance
21	M	Prostate	50s	TP53	N		Stomach	NA	Surveillance
22	M	Colorectal	40s	BRCA2	N	APC p.Ile1307Lys		3B	Surveillance
23	F	Colorectal	50s	BRCA2	N			3B	Surveillance and prophylactic surgery
24	F	Breast	30s	MSH6	N			NA	Surveillance
25	M	Pancreas	60s	STK11	Y			NA	Surveillance
26	F	Breast	60s	FLCN	N			NA	Surveillance
27	M	Prostate	50s	FH	N			NA	Surveillance
28	F	Pancreas	50s	TSC1	Y			3B	Surveillance
29	M	Colorectal	50s	BRCA2	Y		Bladder	3B	Surveillance
30	F	Ovarian	40s	TP53	N		Breast	NA	Surveillance and prophylactic surgery
31	F	Colorectal	40s	BRCA2	N	MUTYH, FH p.Lys477dup		3B	Surveillance and prophylactic surgery
32	F	Breast	40s	PMS2	N			NA	Surveillance
33	M	Colorectal	30s	BRCA2	N			3B	Surveillance
34	F	Colorectal	40s	BRCA2	Y	MUTYH		3B	Surveillance and prophylactic surgery
35	F	Breast	30s	SDHAF2	N			NA	Surveillance
36	F	Colorectal	70s	BRCA2	Y			3B	Surveillance and prophylactic surgery
37	F	Pancreas	50s	FH	N	BRCA2		NA	Surveillance
38	M	Colorectal	70s	BRCA1	N		Eye	3B	Surveillance
39	F	Colorectal	50s	TP53	N			NA	Surveillance and prophylactic surgery
40	F	Breast	50s	BAP1	N			NA	Surveillance
41	F	Colorectal	20s	FH	N			NA	Surveillance
42	F	Breast	40s	DICER1	N			NA	Surveillance
43	F	Colorectal	50s	BRCA1	Y			3B	Surveillance and prophylactic surgery
44	M	Colorectal	40s	PALB2	N			3B	
45	F	Breast	30s	PMS2	N			NA	Surveillance
46	M	Colorectal	40s	BRCA1	N			3B	Surveillance
47	F	Colorectal	50s	BRCA1	N			3B	Surveillance and prophylactic surgery
48	M	Prostate	60s	SDHC	N			NA	Surveillance
49	M	Colorectal	30s	FLCN	N			NA	Surveillance
50	M	Colorectal	50s	BRCA2	N			3B	Surveillance
51	F	Breast	40s	RET	N	BRCA1	Skin	3B	Surveillance
52	M	Colorectal	20s	BRCA1	N	CHEK2, ERCC3		3B	Surveillance
53	F	Breast	50s	MSH6	N		Uterus	NA	Surveillance
54	F	Colorectal	30s	BRCA2	Y	MITF	Breast	3B	Surveillance and prophylactic surgery
55	M	Colorectal	60s	BRCA2	Y			3B	Surveillance
56	M	Colorectal	30s	BRCA1	Y	MLH1		3B	Surveillance
57	M	Colorectal	60s	BAP1	N	PMS2		NA	Surveillance
58	F	Breast	40s	DICER1	N			NA	Surveillance
59	M	Colorectal	40s	PALB2	N			3B	
60	M	Breast	50s	CDKN2A	N			NA	Surveillance
61	F	Ovarian	50s	BAP1	N			NA	Surveillance
62	M	Prostate	50s	DICER1	N			NA	Surveillance
63	F	Colorectal	60s	FH	N			NA	Surveillance
64	M	Colorectal	40s	NF1	Y			3B	Surveillance
65	F	Colorectal	40s	PTCH1	N			NA	
66	M	Colorectal	60s	RET	N			3B	Surveillance
67	M	Colorectal	50s	RET	N			3B	Surveillance
68	F	Colorectal	30s	RB1	Y	APC p.Ile1307Lys	Retinoblastoma	NA	Surveillance
69	M	Colorectal	30s	NF1 (mosaic)	N			3B	Surveillance
				PALB2	N			3B	

*Pt* patient, *Dx* diagnosis, *Hx* history, *F* female, *M* male, *Y* yes, *N* no

Fifty-one patients (74% of patients with high penetrance additional findings) did not meet the current criteria to receive GT for the additional gene identified in eGT. These include patients with P/LP variants identified in *BRCA1*/*BRCA2* (*n* = 9), *MSH6/PMS2* (*n* = 7), *FLCN* (*n* = 4), *SDHB/SDHC/SDHAF2* (*n* = 3), *TP53* (*n* = 3), *BAP1* (*n* = 3), *CDKN2A* (*n* = 3), *DICER1* (*n* = 3), *PALB2* (*n* = 3), *RET* (*n* = 3), *CDH1* (*n* = 1), *ETV6* (*n* = 1), *PTCH1* (*n* = 1), *VHL* (*n* = 1), and *NF1* (*n* = 1 (mosaic)). Additionally, six patients had *FH* P/LP variants (p.Gln376Pro (*n* = 3), p.His402Tyr (*n* = 2), p.Gly397Arg (*n* = 1)) that have been reported in homozygous and compound heterozygous patients with fumarate hydratase deficiency, but have not, to our knowledge, been reported in patients with HLRCC. Five of the six patients with these variants had no known features of HLRCC and one of them had uterine fibroids. Therefore, although these variants were classified as P/LP for AR fumarate hydratase deficiency, whether they confer increased risk for HLRCC is currently uncertain.

### Variants of uncertain significance (VUSs) identified in eGT

One of the main concerns restricting the use of eGT is the potential burden of assessing VUSs by laboratories performing the test. To understand the impact of VUSs in variant interpretation and reporting processes of eGT, we analyzed the number of variants classified as VUS in patients with one of the five common cancer types. Overall, 57% (5238/9187) of the patients had at least one VUS identified, with 56.8% (1275/2243), 59.4% (1223/2060), 52.5% (589/1122), 54.5% (898/1648), and 59.3% (1253/2114) of breast, colorectal, ovarian, pancreatic, and prostate cancer patients having at least one VUS, respectively. The number of VUSs identified ranged from zero to nine, with a median of one VUS per patient.

## Discussion

Our analyses on 17,523 patients with solid tumors revealed that eGT would be beneficial for individuals with many cancer types, including those who do not frequently receive GT in the current practice. In the present study, 16.7% of patients had at least one P/LP variant in cancer susceptibility genes, which is higher than 13.3% reported recently by Samadder et al. in 2984 cancer patients [64]. Differences observed in positivity rates may be due to varying proportions of cancer types in two cohorts, patient populations at different cancer care institutions, possible biases in the referral of patients, and differences in sequencing assays and analysis pipelines. While our current study was performed in the context of concurrent tumor-normal sequencing, the overall rate of germline P/LP variants detected here is lower than the 30.6% ratio previously reported in patients who underwent germline testing following tumor sequencing [65], consistent with observations that follow-up germline testing after tumor sequencing may be preferentially performed for patients with the highest level of suspicion for having hereditary cancer predisposition and may be underused for others, as proposed by the authors [65].

Our results are consistent with prior observations that a significant proportion of patients with hereditary cancer predisposition were not detected by guideline-based GT models employed at that time [15, 64, 66] and also suggest that eGT, compared to current multigene panels, can identify some patients at high risk to develop other cancers in the future. These findings would allow opportunities for early surveillance and, in a small subset of cases, prophylactic interventions for patients and their family members, which would not have been detected using currently employed phenotype targeted gene panels. Currently, gene panels targeted for each condition vary widely among different institutions and laboratories. While some groups test a broad range of genes that have been implicated in a cancer type, others may choose to only target genes with high diagnostic yield or restrict testing to patients with specific phenotypes only (i.e., *CDH1* in patients with lobular breast cancer and personal/or family history of gastric cancer, *TP53* in patients who meet Li-Fraumeni syndrome GT criteria, *NF1* in patients with known features of neurofibromatosis type 1, juvenile polyposis syndrome genes such as *BMPR1A* and *SMAD4* in patients with multiple juvenile polyps, or *POLD1* in colorectal cancer patients with demonstrated high mutation burden). However, it has been increasingly recognized that the phenotypic spectrum of cancer genes may be wider than previously recognized and patients may present with mild features that may be missed without thorough clinical evaluation. One group of genes that is typically targeted in a selected manner is Lynch syndrome genes. In the current study, Lynch syndrome was identified in 0.9% of ovarian and 0.8% of prostate cancer patients receiving eGT. Lynch syndrome genes are recently included in GT guidelines for prostate cancer patients. Ovarian cancer patients, however, are typically tested for Lynch syndrome genes only if they have prior personal or family history that meets Lynch syndrome GT criteria, their tumors have endometrioid/clear cell histology, or are demonstrated to harbor MSI and/or mismatch repair (MMR) protein deficiency, although MSI and MMR profiling are not routinely performed for ovarian cancer patients at all institutions. Additionally, four of nine ovarian cancer patients with Lynch syndrome in our study did not have MSI or MMR protein deficiency by IHC. Similarly, in our breast cancer patients, genes that are often only targeted in the presence of additional personal and/or family history, such as *NF1*, *TP53*, and *CDH1*, added a minor increase in the diagnostic yield, but they established a molecular diagnosis for the underlying condition for these patients, providing clinical benefit. In fact, both of the two patients with *CDH1* variants and one of the two patients with *TP53* would have been missed based on the current GT criteria.

There are various reasons for restricting GT to selected genes, including resources needed for laboratories to assess a larger number of genes/variants and pre-/post-test genetic counseling regarding additional findings. For laboratories, the highest impact is expected to be on the increase in the number of variants interpreted post-sequencing. Due to the content overlap in many targeted cancer gene panels and to allow customization, in current practice, clinical laboratories often sequence multiple gene panels using a single probe set and limit the analysis to targeted genes in downstream analyses. Therefore, the benchwork and sequencing costs for a small gene panel are often comparable to those of sequencing larger gene panels, while more variants that require expert review and classification are expected to be uncovered as the number of targeted genes increases. Our results suggest that eGT would identify additional VUSs in a significant portion of patients receiving eGT. VUSs pose various challenges for laboratories, clinicians, and patients. Laboratories may need to perform additional analyses, such as segregation or RNA studies, to help clarify the clinical significance of VUSs and dedicate resources to periodically capture recently published data for reassessing VUSs, which may lead to reclassification [67–70]. VUSs may cause difficulties for clinicians in the risk assessment and counseling of the patients and their family members [71–73] and may also potentially be misinterpreted or lead to increased anxiety for the patients [74–76]. Therefore, the benefits of performing eGT should be weighed against the added number of VUSs identified with this approach.

This study has several limitations. First, as mentioned above, our cohort consisted of patients treated at a large cancer care center, and patients were enrolled in eGT by their referring physicians. Although previously known hereditary predisposition was not an exclusion criterion, there may be physician biases in the enrollment of such patients in the study cohort. Second, although our cohort was unselected for cancer type, age of onset, race/ethnicity, or family history, it consisted of patients who received paired tumor sequencing. Therefore, it was enriched for those undergoing systemic therapy and thus with advanced disease. In the recent study by Samadder et al. [64], the rate of germline findings did not vary based on the patient’s stage of disease and was similar in patients with stage 0–2 and those with stage 3–4 cancer, suggesting that the impact of disease stage on the rate of germline findings may not be substantial, although other factors, such as tumor site, cannot be excluded. Third, our assay has limitations in detecting certain variants such as structural rearrangements, transposon element insertions, and low-level mosaicism, and therefore, the occurrence of such variants cannot be excluded. Finally, gene-disease associations and genetic testing guidelines are not static, and therefore, the relevance of a gene for a given cancer type and whether an individual meets the GT criteria for a specific gene may change over time.

The widespread use of multigene panels and the expansion in preventative and treatment implications of germline findings have raised a question on whether universal genetic testing should be offered to all cancer patients [16, 49, 64, 77]. The results of our study support that expanding patient and gene selection criteria for hereditary cancer predisposition testing would identify actionable findings and provide clinical benefit for larger groups of cancer patients and their families. Our findings demonstrate that in both more common and in rare cancer types, a substantial proportion of individuals in our cohort carried germline variants conferring cancer susceptibility. Since this study was performed at a large cancer referral hospital, studies on the yield of eGT in patients treated at community hospitals and clinics and larger cohorts of patients with rare cancer types will help better understand whether these results would be more broadly representative. Certainly, clinical outcomes in carriers identified via eGT, risks associated with discovery of uncertain findings, availability of appropriate care following testing, and cost-benefit analyses will also need to be considered to fully understand the feasibility and utility of an eGT approach. It should also be noted that as the number of germline alterations associated with therapeutic implications increases, the importance of identifying carriers of these germline pathogenic variants will become even more critical for proper clinical management.

## Conclusions

eGT can identify hereditary cancer predisposition in patients with a broad range of solid tumors, which would not have been detected by current guideline-based GT models, including findings that indicate a high risk to develop other cancers in the future. Therefore, eGT can allow increased opportunities for cancer surveillance and intervention for patients and their at-risk family members, as compared to traditional targeted gene panel testing approaches.

## Supplementary Information

**Additional file 1: Table S1.** Genes tested on GERMLINE MSK-IMPACT test.

**Additional file 2: Table S2.** Cancer surveillance and prophylactic surgery recommendations referred to for determining actionability of findings identified in eGT.

**Additional file 3: Table S3.** Genes tested on MSK-IMPACT grouped based on their penetrance and inheritance type.

**Additional file 4: Fig. S1.** Comparison of the cancer type incidence rates in the study cohort to the incidence rates reported by the National Cancer Institute Surveillance, Epidemiology, and End Results program.

## Data Availability

All de-identified genomic results for the patients in this study are available in the cBioPortal for Cancer Genomics [78, 79] at http://cbioportal.org/msk-impact.
