# Supplementary material for: Diagnostic yield and clinical relevance of expanded genetic testing for cancer patients
Source: Genome Med. 2022 Aug 15;14:92. doi: 10.1186/s13073-022-01101-2 (PMC9377129; doi:10.1186/s13073-022-01101-2)
Supplement: Supplementary file 4 — Additional file 4: Fig. S1. Comparison of the cancer type incidence rates in the study cohort to the incidence rates reported by the National Cancer Institute Surveillance, Epidemiology, and End Results program. [file 13073_2022_1101_MOESM4_ESM.docx]

**Figure S1:** Comparison of the cancer type incidence rates in the study cohort to the incidence rates reported by the National Cancer Institute Surveillance, Epidemiology, and End Results program.
